# The role of Indigenous and Community Conservation Areas in herpetofauna conservation: a preliminary list for Santa Cruz Tepetotutla, Oaxaca Mexico

**DOI:** 10.3897/zookeys.1029.62205

**Published:** 2021-04-08

**Authors:** Pablo Rogelio Simón-Salvador, Medardo Arreortúa, Carlos A. Flores, Hermes Santiago-Dionicio, Edna González-Bernal

**Affiliations:** 1 Instituto Politécnico Nacional. Centro Interdisciplinario de Investigación para el Desarrollo Integral Regional, Unidad Oaxaca. Laboratorio de Ecología de Anfibios (ECA). Hornos 1003, Col. Noche Buena, 71230, Santa Cruz Xoxocotlán, Oaxaca, México Instituto Politécnico Nacional Oaxaca Mexico; 2 CONACYT – Instituto Politécnico Nacional. Centro Interdisciplinario de Investigación para el Desarrollo Integral Regional, Unidad Oaxaca. Laboratorio de Ecología de Anfibios (ECA). Hornos 1003, Col. Noche Buena, 71230, Santa Cruz Xoxocotlán, Oaxaca, México Instituto Politécnico Nacional Oaxaca Mexico

**Keywords:** Amphibians, endemic, ICCAs, montane cloud forest, rediscovery, reptiles

## Abstract

The montane cloud forests of the Sierra Madre de Oaxaca (SMO) host a remarkable herpetofauna diversity and represent one of the most important areas of endemism for Mexico and Mesoamerica. Although the area has been previously studied, most of the extant records for this group are biased to locations accessed by paved roads. In addition, an important proportion of this territory is conserved by Indigenous and Community Conservation Areas (ICCA), but little information of the species occurring within these areas exists. Therefore, information on the distribution of many endemic taxa in this region to date is either underestimated or incomplete. With the aim of increasing the ecological and distributional knowledge of this group in remote areas, we carried out field surveys in Santa Cruz Tepetotutla Oaxaca, a locality 25 km in a straight line to the closest paved road that conserves 9,670 ha of land through the ICCAs modality. Surveys were made during 2018 and 2019, including both dry and wet seasons. A total of 40 species of amphibians and reptiles were recorded: 32.5% of these records represent distributional range extensions, while 20% represent altitudinal range extensions. A total of 17.5% are records of species under a high risk category, highlighting both the relevance of studying remote areas to increase species population knowledge and the role of community conservation actions for species persistence. Finally, our records include the rediscovery of *Rhadinella schistosa*, a species undetected for more than 50 years.

## Introduction

Currently the planet is experiencing a well-documented biodiversity crisis where amphibians and reptiles are the two most threatened vertebrate groups (Hoffmann et al. 2010). Most of their declines have been recorded in the Neotropics where species with restricted distributions and high degree of endemism occur and also where habitat loss, climate change, introduced species and diseases are a constant threat (Brooks et al. 2002; Whitfield et al. 2007; Whitfield et al. 2014).

Nowadays, indigenous communities play an important role in the conservation of biodiversity because they have a direct relationship with local ecosystems by exploiting its resources at the same time as establishing community conservation programmes that allow the protection of flora and fauna species (Gadgil et al. 1993). Worldwide, indigenous people own 37.9 million km^2^ of land, of which 7.8 million km^2^ are within protected areas, representing 40% of protected areas worldwide (Garnett et al. 2018).

Within the Neotropics, Mexico is identified as a country that harbours great herpetofauna richness with 1292 recognised species (394 amphibians and 898 reptiles) (Johnson et al. 2017). In the case of amphibians, more than 60% of species are endemic, but not all occur within federal protected natural areas; however, 73% of endemic species and 26% of micro-endemic species are represented in social conservation areas, whether private or communal (Ochoa-Ochoa 2009). Within the country, the State of Oaxaca ranks first in the number of registered species (327 reptiles and 150 amphibians) (Flores Villela and García-Vázquez 2014) with a large amount of endemic species as a result of its complex topography and great variety of climates (Casas-Andreu et al. 1996, 2004; Mata-Silva et al. 2015). Oaxaca is also the State with the largest surface area under the Indigenous and Community Conservation Areas (ICCA) system, with a total of 1296.90 km^2^, representing 23.36% of the ICCA’s area nationwide (García-Mendoza et al. 2014; Mata-Silva et al. 2015; CONANP 2020). Many of these areas are immersed in the Chinantla, Mazateca, Mixe and Zoque ethnic regions, located many kilometres away from paved roads (CONANP 2020).

Despite being a relatively small area, the physiographic sub-province known as Sierra Madre de Oaxaca (SMO) (Ortiz-Perez et al. 2014), stands out for having the highest amount of endemic species of herpetofauna for Mexico and Oaxaca (Mata-Silva et al. 2015), with 31% of these endemic species in some category of extinction risk (IUCN 2019). Most of these species are associated with montane cloud forests and the region is considered a priority area for amphibian and reptile conservation (Lamoreux et al. 2015).

For threatened species, increasing distributional records is of paramount importance to model their potential distribution, to propose conservation zones that effectively cover their areas of occurrence and to understand the role that ICCAs play in their survival in addition to providing baseline information for ecological studies at these sites (Duran et al. 2012; Nelson 2012; Soto-Huerta and Clause 2017; Reyes-Velasco and Ramírez-Chaparro 2019; Clause et al. 2020). However, distributional knowledge of some endemic species in this region is still incomplete. This is mainly due to two phenomena: 1) a low number of herpetological studies in the area (Caldwell 1974; Lips et al. 2004; Delia et al. 2013; Caviedes-Solis et al. 2014; Furbush et al. 2017) and 2) biased sampling restricted to easily accessible areas, such as along paved roads (Guedes et al. 2017; Barends et al. 2020).

These aspects contribute to a disparity in terms of herpetofauna richness and the amount of ecological and natural history data for species in highly diverse areas, such as the Neotropics (Urbina-Cardona 2008b). The Neotropics harbour a high proportion of the world’s herpetofauna; however, effective conservation plans in the area are scarce due to a lack of basic biological information that could lead to ecological studies (Tapley et al. 2018). In this sense, generating species inventories gains relevance as they represent the most basic information required to lead to any biological research (Silveira et al. 2010). When considering that this zone harbours around 50% of herpetofauna species, but also is under constant human pressure, conducting surveys to improve known species occurrence represents the first step to reach multidisciplinary research that can improve herpetofauna conservation.

In general, herpetofauna studies in the tropics, such as Mesoamerica, are limited (Soto-Huerta and Clause 2017). Even though the SMO is one of the relatively highly studied areas in the region, several enigmatic species are still only known from their type locality or nearby localities: *Anolis rubiginosus*, *Abronia mitchelli*, *Cryophis hallbergi*, *Craugastor polymniae*, *Quilticohyla acrochorda*, *Sarcohyla cyanomma* and *Sarcohyla calvicollina* (IUCN 2020). Moreover, the fact that most of the knowledge is concentrated at localities in close proximity to paved roads leaves most of the territory with information gaps (Casas-Andreu 2004; Soto-Huerta and Clause et al. 2017). In addition, new species descriptions for this region indicate that Oaxaca’s biodiversity is still underestimated (Mata-Silva et al. 2015). With the aim of increasing the herpetological knowledge of the SMO, both at remote areas and at community conservation areas (ICCAs), we explored a relatively pristine cloud forest in Santa Cruz Tepetotutla.

In this paper, we provide herpetofauna occurrence data at a locality 25 km in a straight line from the closest paved road, highlighting the importance of communal conservation actions to ensure the existence of endangered species. The 40 species included in this list represent 9% of the herpetofauna species reported for Oaxaca (444 species), of which 11 represent 5% of the endemic species reported for the SMO physiographic sub-province (217 species; Mata-Silva et al. 2015; Canseco-Márquez et al. 2017; Johnson et al. 2017). Amongst our records, we present the rediscovery of a species undetected for more than 50 years. We consider that this type of information contributes to fill biological gaps in this highly diverse area.

## Material and methods

### Study area

**Figure 1.**
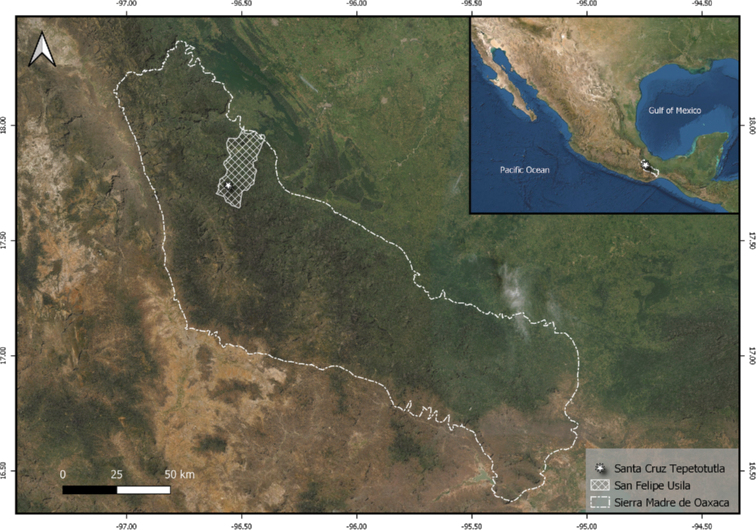
Santa Cruz Tepetotutla locality in the Chinantla region on the northern slopes of the Sierra Juarez, Oaxaca Mexico.

Santa Cruz Tepetotutla (17.7391°N, -96.5582°W) is located in the Chinantla Region on the northern slopes of the Sierra Juarez, in the physiographic Province known as the Sierra Madre de Oaxaca (SMO) Mexico (García-Mendoza 2004) (Fig. 1). In this Region, the tropical montane cloud forest is distributed between 700 and 2500 m a.s.l. (Flores and Manzanero 1999). Since 2004, the local community of this region has preserved 9,670 ha of montane cloud forest under the Indigenous and Community Conserved Area (ICCAs) modality, officially recognised and certified by the National Protected Area Commission in Mexico (CONANP). People manage their territory by having specific sites for subsistence activities, such as agriculture (corn and coffee plantations) and cattle paddocks that are continuously rotated to avoid the deforestation of new areas (Pedro Osorio-Hernandez, pers. comm.). These social conservation actions make this forest one of the largest preserved patches of this ecosystem in the country (Ponce-Reyes et al. 2012). The vegetation in conserved areas is dominated by species, such as *Pleuranthodentron lindenni*, *Clethra integerrima*, *Miconia trinervia*, *Oreomunnea mexicana* and *Ticodendron incognitum* (Rincon 2007). At disturbed areas, *Liquidambar macrophylla* and *Pinus chiapensis* are dominant species. The access to the community was limited to pathways until 2004 when an unpaved road for motor vehicles was opened.

### Fieldwork

We conducted surveys during 2018 and 2019 during both the dry and wet seasons, within a pristine cloud forest and managed sites from 800 m a.s.l. to above 2,200 m a.s.l. The monitoring was divided into three schedules throughout the day (9 a.m. to 1 p.m., 3 p.m. to 7 p.m. and from 9 p.m. to 1 a.m.) covering a total of 12 hours by four persons, during 11 field trips with a duration of five days each. To ensure we obtained records for species that have different activity patterns and habitat use, we carried out diurnal and nocturnal surveys in forest gaps, crops, streams and primary forest, searching microhabitats where herpetofauna take refuge, such as beneath rocks, inside burrows, under fallen logs etc.

Every time an organism was encountered, its biometric data and geographic location were recorded using a GPS device (Garmin 64^st^) with 5 m error range. All organisms were photographed and released afterwards.

We monitored herpetofauna in montane cloud forest with different management conditions: 1) disturbed: sites located adjacent to agriculture or cattle-raising areas, 2) recovered: sites located in recovered cloud forest near to roads and paths and 3) undisturbed areas: sites located within primary cloud forest located more than 1,500 m in an aerial straight line from the nearest anthropogenic disturbance.

### Laboratory work

A literature review was carried out to determine any distributional range extension, either altitudinal or geographic. For this purpose, we also used the historical records available in the CONABIO and GBIF databases (CONABIO 2020; GBIF.org 2020). In order to reduce bias due to identification errors, only presence points of organisms deposited in scientific collections were considered.

We used the “measurement” tool in the Software Quantum GIS version 3.10.6 (Quantum GIS Development Team 2020) to calculate the distance between historical records and our records. Distances greater than 10 km in a straight line and outside the potential distribution proposed by IUCN and CONABIO were considered distributional extensions (IUCN 2019; CONABIO 2020). Additionally, a Species Accumulation Curve (SAC) for 11 field trips was plotted with the R (R Core Team 2020) specaccum function of “vegan” package (Oksanen et al. 2020) and compared with the estimated species richness using the Chao1 estimator obtained with EstimateS (Colwell 2013) and, finally, we created a graphic with the species abundance.

Specimen identification was verified by Luis Canseco-Marquez. Common names suggested by Liner and Casas-Andreu (2008) were followed. All photographs were deposited at the Colección Nacional de Anfibios y Reptiles (CNAR) of the Instituto de Biología, Universidad Nacional Autónoma de Mexico (UNAM) in Mexico City.

## Results

**Figure 2.**
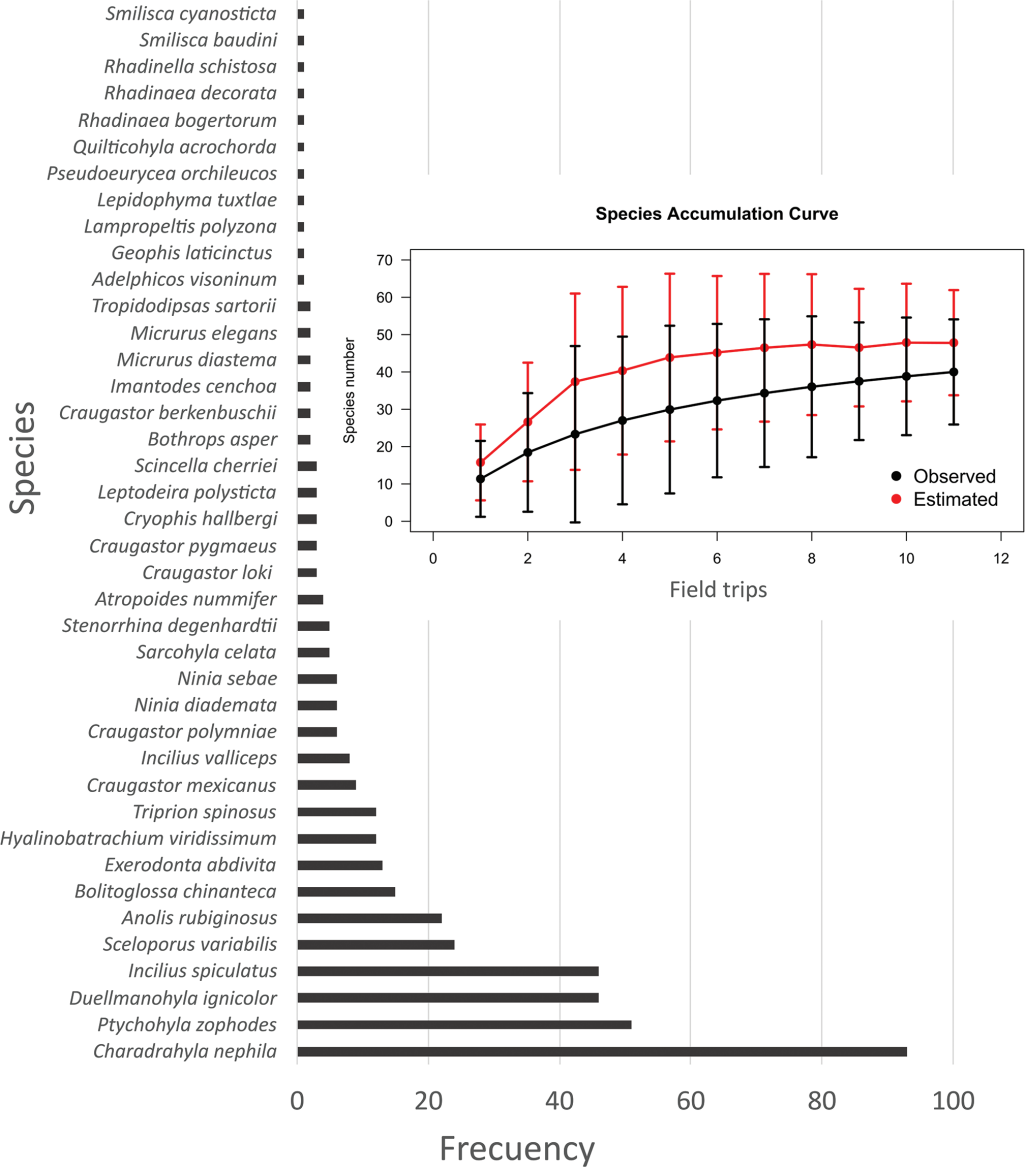
Species accumulation curve and abundance for 40 amphibian and reptile species recorded during 11 field trips. Black line and dots represent the observed data curve, while red line and dots represent the Chao1 estimated curve.

We found a total of 40 species, including two salamanders, seventeen anurans, four lizards and seventeen snakes (https://doi.org/10.15468/utcd2c; Table 1) with a field effort invested of 2,640 effective hours. Even when the species accumulation curve had not reached the asymptote (Fig. 2), when comparing the observed vs. estimated species by the Chao1 index, it was noticeable that the distance between both curves was relatively low, although a greater sampling effort is required. The recorded species represent 18.43% of the total known amphibians and reptiles from the Sierra Madre de Oaxaca physiographic sub-province, 10 of which represent 14% of the endemisms reported for this same sub-province (Mata-Silva et al. 2015). In addition, five of the registered amphibian species (12.5%) are considered at risk (CR = Critically Endangered, EN = Endangered, VU = Vulnerable) (IUCN 2019).

According to the IUCN, of the reported species, 2.5% (*Quilticohyla acrochorda*) are catalogued as critically endangered, 2.5% (*Ptychohyla zophodes*) as vulnerable, 7.5% (3 amphibians) as endangered, 15% (6 amphibians) as near threatened, 60% (8 amphibians and 16 reptiles) as least concern, 10% (4 reptiles) as data deficient and finally one species 2.5% (*Bothrops asper*) has not been evaluated.

In the case of risk categories assigned by the Mexican Government (NOM-059-SEMARNAT-2010), only thirteen of the reported species have been evaluated: three amphibians and ten reptiles. The amphibians *Craugastor polymniae*, *Duellmanohyla ignicolor* and *Craugastor berkenbuschii* are classified as under special protection (Pr), two reptile species (*Metlapilcoatlus nummifer* and *Anolis rubiginosus*) as threatened (A), while the 8 remaining reptile species as under special protection (Pr) (Table 1).

We present new records of *Scincella cherriei* for the SMO, which was previously known only from the southeast of Oaxaca (Mata-Silva et al. 2015). In addition, we provide the first record of *Lepidophyma tuxtlae* for this Municipality, which represents a 23 km range extension from the nearest record and, finally, we report the first record for *Micrurus elegans* for the community of Santa Cruz Tepetotutla (Bezy and Camarillo 2002; Soto-Huerta and Clause 2017).

For 20% of the recorded species (5 amphibians and 3 reptiles), we registered an increase in altitudinal distribution and for 32.5% (8 amphibians and 5 reptiles) an increase in geographical extension (Table 1). Amongst the species for which we recorded new localities, six were known from less than five localities (*Craugastor polymniae*, *Exerodonta abdivita*, *Pseudoeurycea orchileucos*, *Quilticohyla acrochorda*, *Sarcohyla celata* and *Bolitoglossa chinanteca*) (Fig. 2A–F) and three were previously known only from their type locality (*Anolis rubiginosus*, *Cryophis hallbergi* and *Rhadinaea bogertorum*) (Fig. 2G–I); thus, these are considered with limited information (Clause et al. 2016). For these species, we provide brief notes on ecology indicating the relevance of these new records. The information is presented alphabetically and organised by class, family, genus and species.

**Table 1.** List of amphibians and reptiles of Santa Cruz Tepetotutla. * = Distributional range extension, ** = Altitudinal range extension, R = Rediscovery.

Taxa	IUCN Red List Category	EVS	Status NOM-059 SEMARNAT	Altitude (m a.s.l.) recorded in this study	Known altitude m a.s.l.	Endemism	Catalogue number
**AMPHIBIA (19 species)**							
** ANURA **							
**Bufonidae (2 species)**							
Incilius spiculatus**	EN	M (13)	Not included	600–1760	800–1689 (Mendelson 1997)	Oaxaca SMO	IBH-RF 607
*Incilius valliceps*	LC	L (6)	Not included	880	0–1800 (Kenneth 1970)	Not endemic	IBH-RF 608
**Centrolenidae (1)**							
*Hyalinobatrachium viridissimum*	LC	M (10)	Not included	1250	20–1275 (Mendoza-Henao et al. 2020)	Not endemic	IBH-RF 605
**Craugastoridae (5 species)**							
*Craugastor mexicanus*	LC	H (16)	Not included	1540	700–3420 (Mexico Red List Assessment Workshop 2019)	Mexico	IBH-RF 597
Craugastor polymniae*/**	NT	H (18)	Pr	1100	1420–1500 (Campbell et al.1989; Stuart 2008)	Oaxaca SMO	IBH-RF 598
Craugastor pygmaeus*	LC	L (9)	Not included	1550	0–2145 (Ahumada-Carrillo 2013)	Not endemic	IBH-RF 599
*Craugastor berkenbuschii*	LC	H (14)	Pr	1300	200–1900 (Urbina-Cardona 2008)	Mexico	IBH-RF 600
*Craugastor loki*	LC	M (10)	Not included	1510	0–2000 (Lynch 2000)	Not endemic	IBH-RF 631
**Hylidae (9 species)**							
*Charadrahyla nephila*	EN	M (13)	Not included	800–2200	680–2256 (Mendelson and Campbell 1999)	Mexico	IBH-RF 596
Duellmanohyla ignicolor*	NT	H (14)	Pr	1000–1600	680–1850 (Furbush et al. 2017)	Oaxaca SMO	IBH-RF 602
Exerodonta abdivita*	NT	H (15)	Not included	800–1250	89–1600 (Campbell 2000; Delia et al. 2013 and Ramírez-González et al. 2014)	Mexico	IBH-RF 603
Ptychohyla zophodes**	VU	M (13)	Not included	800–1650	400–1500 (Campbell 2000)	Oaxaca SMO	IBH-RF 617
*Quilticohyla acrochorda*	CR	H (14)	Not included	800	594–900 (Campbell 2000).	Oaxaca SMO	IBH-RF 618
Sarcohyla celata*/**	NT	H (14)	Not included	2210	2559–2890 (Caviedes-Solis pers. com.; Duellman 2001)	Oaxaca SMO	IBH-RF 622
Smilisca cyanosticta*	LC	M (12)	Not included	1200	300–1200 (Stuart 2008)	Not endemic	IBH-RF 626
*Smilisca baudini*	LC	L (3)	Not included	1200	300–1200 (Stuart 2008)	Not endemic	IBH-RF 625
*Triprion spinosus*	NT	H (14)	Not included	1200–1500	95–2000 (Santos Barrera et al. 2008)	Not endemic	IBH-RF 628
** CAUDATA **							
**Plethodontidae (2 species)**							
Bolitoglossa chinanteca*/**	NT	H (18)	Not Included	1000–1500	1500 (Rovito et al. 2012)	Oaxaca SMO	IBH-RF 594
Pseudoeurycea orchileucos*	EN	H (18)	Not included	1200	800–1390 (Brodie et al. 2002)	Oaxaca SMO	IBH-RF 616
**REPTILIA (21 species)**							
** SQUAMATA **							
**Dactyloidae (1 species)**							
Anolis rubiginosus*/**	DD	H (16)	A	1000–1500	1768–1900 (Bocourt 1873)	Oaxaca SMO	IBH-RF 592
**Phrynosomatidae (1 species)**							
*Sceloporus variabilis*	LC	L (5)	Not included	1000–1200	0–2500 (IUCN 2020)	Not endemic	IBH-RF 623
**Scincidae (1 species)**							
*Scincella cherriei* *	LC	M (12)	Not included	1100–1200	0–1860 (Chaves et al. 2013)	Not endemic	IBH-RF 624
**Xantusiidae (1 species)**							
Lepidophyma tuxtlae*	DD	H (16)	A	1280	0–1500 (Bezy and Camarillo 2002)	Mexico	IBH-RF 610
**Colubridae (2 species)**							
*Lampropeltis polyzona*	LC	M (11)	Not included	1120	0–3000 (Heimes 2016)	Not endemic	IBH-RF 609
*Stenorrhina degenhardtii*	LC	L (9)	Not included	1230	0–2800 (IUCN 2020)	Not endemic	IBH-RF 627
**Dipsadidae (11 species)**							
Adelphicos visoninum*	LC	M (10)	Pr	1100–1300	0–1600 (Heimes 2016)	Not endemic	IBH-RF 591
*Cryophis hallbergi* */**	DD	H (14)	Not included	1100	1200–2000 (Canseco-Márquez 2007; Heimes 2016)	Oaxaca SMO	IBH-RF 601
*Geophis laticinctus*	LC	M (11)	Pr	1080	730–1800 (Heimes 2016)	Mexico	IBH-RF 604
*Imantodes cenchoa*	LC	L (6)	Pr	1230	0–1700 (Arzamendia et al. 2019)	Not endemic	IBH-RF 606
*Leptodeira polysticta*	LC	Not evaluated	Not included	1210	0–2500 (Heimes 2016)	Not endemic	IBH-RF 611
*Ninia diademata*	LC	L (9)	Not included	1510	0–2438 (Lee et al. 2013; Heimes 2016)	Not endemic	IBH-RF 614
*Ninia sebae*	LC	L (5)	Not included	1180	0–2200 (Chaves et al. 2013; Heimes 2016)	Not endemic	IBH-RF 615
*Rhadinaea bogertorum* */**	DD	H (16)	Pr	1500	2000–2400 (Heimes 2016)	Oaxaca SMO	IBH-RF 619
*Rhadinaea decorata*	LC	L (9)	Not included	1040	0–1200 (Heimes 2016; Chaves et al. 2017)	Not endemic	IBH-RF 620
*Rhadinella schistosa^R^*	LC	13	Pr	1250	800–1600 (Heimes 2016)	Mexico	IBH-RF 621
*Tropidodipsas sartorii*	LC	L (9)	Pr	1450	0–2438 (Heimes 2016)	Not endemic	IBH-RF 629
**Elapidae (2 species)**							
*Micrurus elegans*	LC	M (13)	Pr	1200	100–1700 (Soto-Huerta and Clause 2017)	Not endemic	IBH-RF 613
*Micrurus diastema*	LC	L (8)	Pr	1200	0–1800 (Acevedo et al. 2013)	Not endemic	IBH-RF 612
**Viperidae (2 species)**							
Metlapilcuatlus (Atropoides) nummifer	LC	M (13)	A	1334	670–1800 (Heimes 2016)	Mexico	IBH-RF 593
*Bothrops asper*	Not included	M (12)	Not included	1546	0–2640 (Wallach et al. 2014)	Not endemic	IBH-RF 595

### Amphibia

#### Craugastoridae

***Craugastor polymniae* (Campbell, Lamar & Hillis, 1989)**

Sierra Juárez Robber Frog

This species was only known from two specimens collected in July 1983 at 1420 m a.s.l., 0.8 km north of Vista Hermosa (Campbell et al. 1989). In 2000, Lips looked for this species without success (Lamoreux 2015); however, in 2009, Sean Rovito collected an individual at La Esperanza Comaltepec (Amphibiaweb.com 2009) and, in 2013, Flores-Villela collected 21 individuals of this species in the Sierra Mazateca, 12 km north of the type locality (Flores Villela and CONABIO 2020).

Six individuals of *C. polymniae* were recorded during our surveys increasing the known localities of this rare species to four. Our records are made 9 years after the last time the species was seen in the SMO. Except for one specimen, all were found in primary cloud forest outwith human-disturbed areas. All frogs were found within ~ 3 m from streams.

#### Hylidae

***Exerodonta abdivita* (Campbell & Duellman, 2000)**

Rio Aloapam tree frog

This species is endemic to Oaxaca and is only known from four localities: the type locality in the lowlands of Sierra Mazateca, Vista Hermosa (1600 m a.s.l.), San Mateo Yetla (700 m a.s.l.) and Santiago Jocotepec (89 m a.s.l.) (Delia et al. 2013; Ramírez-González et al. 2014). Our records (17.7334°N, -96.5557°W, datum WGS84, elev: 1079 m a.s.l.) represent an extension of its distributional range of 31.85 km NW from its type locality and 25.26 km from other known localities.

In Santa Cruz Tepetotutla, this species inhabits the relative pristine mesic cloud forest and montane cloud forest. Specimens were commonly found at the lower parts of vegetation near to strong current streams at an altitude of 600 to 1300 m a.s.l.

***Quilticohyla acrochorda* (Campbell & Duellman, 2000)**

Warty mountain stream frog

This species has an extremely restricted distribution, known only from two localities from Sierra Juarez: Valle Nacional (Puente Dañado) and San Mateo Yetla, on the Atlantic versant Oaxaca, Mexico (Campbell and Duellman 2000). We report a single individual (17.7453°N, -96.5669°W, datum WGS84, elev: 880 m a.s.l.) extending the distribution of this species 27.16 km NW from its type locality. A single male calling from a leaf of riparian vegetation at around 2 m height was found sharing its habitat with *E. abdivita*.

***Sarcohyla celata* (Toal & Mendelson, 1995)**

Oaxacan tree frog

This species has a restricted distribution and is known only from two localities in Sierra de Juárez: type locality: 0.9 km N of Cerro Pelon and the Municipality of San Pablo Macuiltianguis (Caviedes-Solis et al. 2015). We report a new population located in the extreme south of the Municipality of San Felipe Usila (17.67255°N, -96.55913°W, datum WGS84, elev: 2,210 m a.s.l.) extending the distribution of this species 18.64 km NW from its type locality and expanding its altitudinal range 345 metres lower than the previously-known range (2559–2890 m a.s.l.). *Sarcohyla celata* individuals were found on vegetation next to a stream in primary cloud forest more than 1,500 m in a straight line from human populations.

#### Plethodontidae

***Bolitoglossa chinanteca* (Rovito, Parra-Olea, Lee & Wake, 2012)**

Chinantec Salamander

This species is only known from three localities in northern Oaxaca: Vista Hermosa Municipality of Santiago Comaltepec, Municipality of San Pedro Yolox along Highway 175 (at approximately 1500 m elevation) and Santiago Zacatepec from the Sierra Mixe (Rovito et al. 2012). We report a new population located in the extreme south of the Municipality of San Felipe Usila (17.7251°N, -96.5590°W, datum WGS84, elev: 1506 m a.s.l.) extending the distribution of this species 15 km NW from the closest locality San Pedro Yolox. The population at this locality seems to be relatively abundant. We found seven individuals perching on the leaves of ferns adjacent to streams both in primary montane cloud forest and in disturbed areas surrounded by coffee and banana plantations.

**Figure 3.**
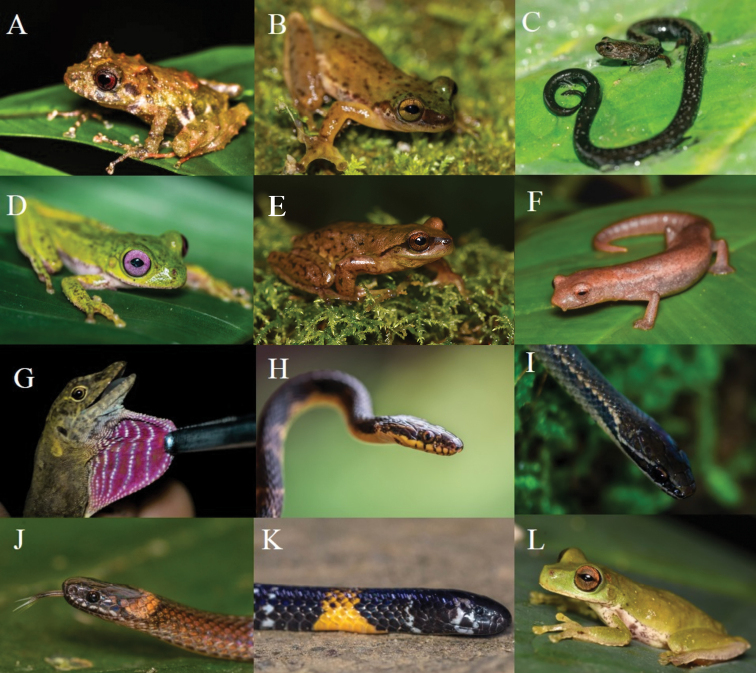
Amphibians and reptiles from Santa Cruz Tepetotutla: **A***Craugastor polymniae***B***Exerodonta abdivita***C***Pseudoeurycea orchileucos***D***Quilticohyla acrochorda***E***Sarcohyla celata***F***Bolitoglossa chinanteca***G***Anolis rubiginosus***H***Cryophis hallbergii***I***Rhadinaea bogertorum***J***Geophis laticinctus albiventris***K***Micrurus elegans* and **L***Duellmanohyla ignicolor*. All photographs by Rogelio Simón-Salvador.

***Pseudoeurycea orchileucos* (Brodie, Mendelson & Campbell, 2002)**

Sierra de Juarez Worm Salamander

This salamander is known only from a few localities on the northern slopes of Sierra de Juarez, Oaxaca, Mexico. Type locality: 5 km San Mateo Yetla and 0.8 km S Vista Hermosa by road (Brodie et al. 2002). The known distribution of this species is expanded 25.73 km W from the closest locality San Mateo Yetla. At 01:00 h PST after heavy rain, we found a single individual on the road (17.73102°N, -96.56056°W, datum WGS84, elev: 1280 m a.s.l.). The area was surrounded by secondary vegetation of montane cloud forest, 400 m from the town and 100 m from the nearest water body. Relative air humidity was 90%.

### Reptiles

#### Dactyloidae

***Anolis rubiginosus* (Bocourt, 1873)**

Sierra Juarez Anole

This rare species is only known from its type locality 9.9 km S of Vista Hermosa, Santiago Comaltepec, Oaxaca. We report the second known population for this species. (17.7332°N, -96.5556°W, datum WGS84, elev: 1000–1500 m a.s.l.) expanding its distribution 26.1 km north of the type locality. The population at this locality seems to be abundant; the individuals were always found perching on the leaves of ferns or herbaceous plants very close to streams immersed in both primary montane cloud forest and around recovered cloud forest.

#### Dipsadidae

***Cryophis hallbergi* (Bogert & Duellman, 1963)**

Hallberg’s Cloud Forest Snake

This micro endemic snake is only known from eight specimens collected 0.6 km south of Campamento Vista Hermosa, 1865 m a.s.l. (Bogert and Duellman 1963; Campbell et al. 1989). We recorded this species (17.73703°N, -96.5647°W, datum WGS84, elev: 1100 m a.s.l.), expanding its distribution 21.59 km SE of the type locality. We obtained five punctual records at an altitudinal range of 1100 to 1500 m a.s.l. Individuals were always found perched on vegetation near streams in conserved montane cloud forest with canopy coverage greater than 80%.

**Figure 4.**
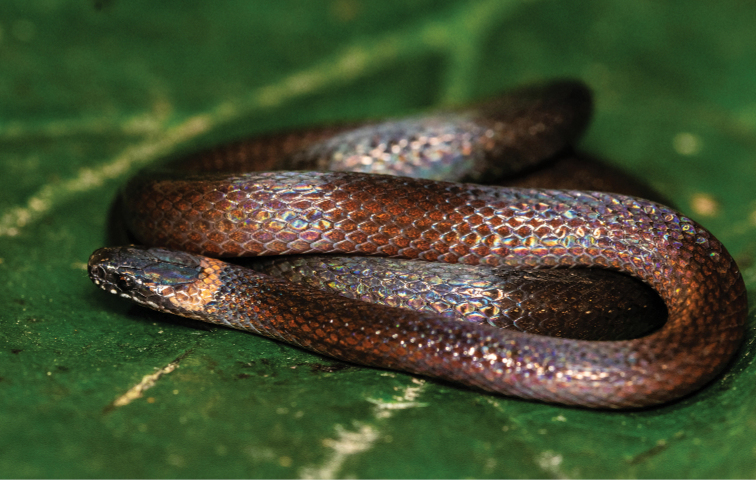
Individual of *Rhadinella schistosa*. Rediscovered in the community of Santa Cruz Tepetotula.

***Rhadinaea bogertorum* (Myers, 1974)**

Oaxacan Graceful Brown Snake

This snake is only known from its type locality 16.8 km N by road from Cerro Pelon, Municipality of Santiago Comaltepec at elevations of 2,025 to 2,075 m a.s.l. (Myers 1974; Heimes 2016). We increased the distribution of this species 21.41 km north of its type locality (17.7249°N, -96.5589°W, datum WGS84, elev: 1510 m a.s.l.). A single individual was found active at dusk in conserved cloud forest with canopy cover over 90%.

#### Rediscovery

***Rhadinella schistosa* (Smith, 1941)**

Brokencollar Graceful Brown Snake

*Rhadinella schistosa* was described, based on seven individuals collected in Cuautlapan Veracruz (Smith 1941). Since then, it was not recorded until 1969 when a specimen (CUM 39790) was found in Vista Hermosa, Municipality of Santiago Comaltepec, 80 km southeast of the type locality. After going undetected for 50 years, we found an adult male of *Rhadinella schistosa* on a road in cloud forest in Santa Cruz Tepetotutla (17.7305°N, -96.5583°W; datum WGS84, elev: 1250 m a.s.l. Catalogue number: IBH-R 621) (Fig. 2). The individual was found during the dry season at 06:50 h PST.

This species is considered under special protection by the Mexican Government (NOM-059 SEMARNAT-2010), but classified as Least Concern by the IUCN, despite the fact that its range is probably less than 20,000 km² (Canseco-Márquez et al. 2007) and very few individuals have been seen.

## Discussion

We provide the first herpetofaunal checklist for Santa Cruz Tepetotutla located in the Chinantla Region. Our results reveal that, within this territory, covering approximately 11,000 ha, occurs a large proportion of endemic herpetofaunal species known for the SMO: 36% of amphibians and 14% of reptiles. In addition, it is inhabited by a high proportion of species listed as high-risk categories according to the IUCN, highlighting the critical role of large patches of relatively pristine vegetation conserved by community actions to host endangered species.

In this study, 30% of the recorded species (5 reptiles and 7 amphibians) were found in areas with conserved cloud forest and patches with slight anthropogenic disturbance. However, five species of anurans (*Craugastor loki*, *C. mexicanus*, *C. polymniae*, *Sarcohyla celata* and *Charadrahyla nephila*) were exclusively observed in areas with primary vegetation. The presence of these species in conserved sites may be related to their intrinsic characteristics, such as foraging habits or reproductive behaviour (Reed and Shine 2002; Gardner et al. 2007; Suazo-Ortuño et al. 2008; Whitfield et al. 2014). For example, members of Craugastoridae are known to depend on dense leaf litter to reproduce; the existence of trees with dense foliage at conserved sites ensures the availability of appropriate microhabitat for their reproduction (Becker et al. 2007). However, this dependence to forest patches with specific characteristics of humidity and litter density increases their vulnerability to habitat reduction. In addition to being exclusive to primary forest, *Sarcohyla celata* and *Charadrahyla nephila* were recorded at streams with no or very low human access, suggesting that they may be even more sensitive to human presence (Urbina-Cardona and Pérez-Torres 2002; Lips et al. 2003). Research is needed to understand their sensitivity to anthropogenic effects.

The description and delimitation of a species’ geographic distribution is one of the main requirements to propose conservation measures; however, sampling bias is a common problem for reliable delimitation (Barends et al. 2020). In general, for rare species, many of which are under extinction risk, these biases often result in information gaps and underestimation of distribution (Costa et al. 2010; Fourcade et al. 2014). With the aim of improving species’ known ranges, we also highlight the importance of increasing efforts to survey remote areas to obtain reliable geospatial data on rare species. Amongst our records, we provide the second known locality for *Anolis rubiginosus*, *Rhadinaea bogertorum* and *Cryophis hallbergi*. It is also important to mention the cases of *Micrurus elegans*, for which, until 2017, only five recorded localities in Oaxaca existed, none of which corresponds to the community of Santa Cruz Tepetotutla (Campbell and Lamar 2004; Soto-Huerta and Clause 2017) and *Scincella cherriei* which was previously recorded only for the physiographic sub-provinces of “Planicie Costera del Golfo” (PCG) and “Depresion Istmica de Tehuantepec” (DIT) in Oaxaca (Mata-Silva et al. 2015) more than 77 km north from our record. In addition, the new geographical information added to the previously-known distribution (Heimes 2016) of the rediscovered *Rhadinella schistosa* suggests a wider distribution area for this species, extending from the northern slopes of Sierra Juarez to the Planicie Costera del Golfo.

Our records also include new altitudinal information for: *Incilius spiculatus*, *Craugastor polymniae*, *Sarcohyla celata*, *Ptychohyla zophodes*, *Bolitoglossa chinanteca*, *Anolis rubiginosus*, *Cryophis hallbergi* and *Rhadinaea bogertorum*. Delimiting accurate altitudinal species’ distribution is gaining relevance because many amphibian and reptile species expand or retract their altitudinal range as a consequence of climate change (Walther et al. 2002; Feldmeier et al. 2020). Usually, these individuals migrate to areas where they can access their required thermal conditions those species with narrow altitudinal distributions, in particular those that occur at high altitudes, under more pressure (Broennimann et al. 2006; Subba et al. 2018; Cordier 2019; Enriquez-Urzelai et al. 2019). These processes are occurring around the world, especially in mountainous areas that also host a great diversity of endemic species (Bergl et al. 2007; Cordier 2019). Amongst our records, *Sarcohyla celata* occurs over a narrow altitudinal range: 2500–2800 m a.s.l. (Duellman 2001; Caviedes-Solis pers. comm.), which makes it particularly vulnerable to climate change.

We emphasise the importance of increasing research for species considered as data deficient (DD). For species identified in this study, *Anolis rubiginosus* is abundant in this locality which would allow the implementation of studies to increase information about its ecology and biology. In case of *Lepidophyma tuxtlae*, we note the need to update the distribution maps generated by the IUCN, since databases, such as GBIF, include records that these maps do not. Perhaps the most extreme cases for species in this category are *Cryophis hallbergi* and *Rhadinaea bogertorum*, of which knowledge about their natural history and ecology is almost non-existent since the only studies on these species are taxonomic (Bogert and Duellman 1963; Myers 1974; Mulcahy et al. 2007, 2011; Ramirez-Bautista et al. 1998). In addition, *Bothrops asper* has not, as yet, been included in any category by the IUCN (Cisneros-Heredia and Jean-Marc 2004; Batista et al. 2020).

Although Oaxaca harbours the greatest herpetofauna diversity in Mexico, the lack of exploration at remote areas has resulted in considerable information gaps (Casas-Andrew 2004; Mata-Silva et al. 2015: Soto-Huerta and Clause 2017). As a consequence, some of the largest forest patches that prevail in the State due to conservation actions carried out by indigenous communities are understudied, which in turn promotes the idea that many amphibian and reptile species occur only in unprotected areas (i.e. Santos-Barrera 2004; Santos-Barrera and Canseco-Márquez 2004; Canseco-Márquez 2007; IUCN SSC 2020). In this context and within the current amphibian and reptile crisis (Gibbons et al. 2000), it is vitally important to highlight the role that indigenous communities play in the conservation of herpetofauna and the way in which these communities contribute to this process. Scientific systematic monitoring in these areas can contribute to understanding their conservation effectiveness by confirming species presence at these sites, as well as providing species inventories essential for their communal land use and regulation plan. Additionally, the information generated can support the access to payments for environmental services which, in this Region, has contributed to turning ICCAs into successful and sustainable participatory models (Mendez-Lopez 2014).

These community conservation schemes have shown to be sustainable management models as low-impact supply and ecotourism areas, as well as delimited zones with low human access that allows the permanence of “intact” space for other species to use, are selectively designated (Boege 2008; Duran et al. 2012). Since they constitute a more inclusive conservation model, they allow indigenous communities to self-manage their natural resources under their own cultural view, as well as social aspirations (Mendez-Lopez 2014). Working together with scientists can help enhance their conservation actions, as well as complementing their decision-making with biological and ecological criteria (Herrera-Flores et al. 2019). In addition, these types of community efforts must be recognised and supported by government agencies with the aim of promoting their replication by other communities with similar social structures. This study demonstrates that ICCAs can play an important role in species conservation and highlights the need to expand scientific systematic monitoring in these areas to understand their conservation role and species composition within them.

## Supplementary materials

Supplementary material 1 Linked data table for The role of Indigenous and Community Conservation Areas in herpetofauna conservation: a preliminary list for Santa Cruz Tepetotutla, Oaxaca México Data type occurrence Explanation note Amphibian and reptile occurrence data in the locality of Santa Cruz Tepetotutla. https://binary.pensoft.net/file/529835This dataset is made available under the Open Database License (http://opendatacommons.org/licenses/odbl/1.0/). The Open Database License (ODbL) is a license agreement intended to allow users to freely share, modify, and use this Dataset while maintaining this same freedom for others, provided that the original source and author(s) are credited.Pablo Rogelio Simón-Salvador, Medardo Arreortúa, Carlos A. Flores, Hermes Santiago-Dionicio and Edna González-Bernal
